# Supplementary material for: A combination strategy targeting enhancer plasticity exerts synergistic lethality against BETi-resistant leukemia cells
Source: Nat Commun. 2020 Feb 6;11:740. doi: 10.1038/s41467-020-14604-6 (PMC7005144; doi:10.1038/s41467-020-14604-6)
Supplement: Supplementary file 2 — Supplementary Information [file 41467_2020_14604_MOESM2_ESM.pdf]

Supplementary Information for

A combination strategy targeting enhancer plasticity exerts synergistic lethality against BETi-resistant leukemia cells

Guo et al.

Supplementary Figure 1

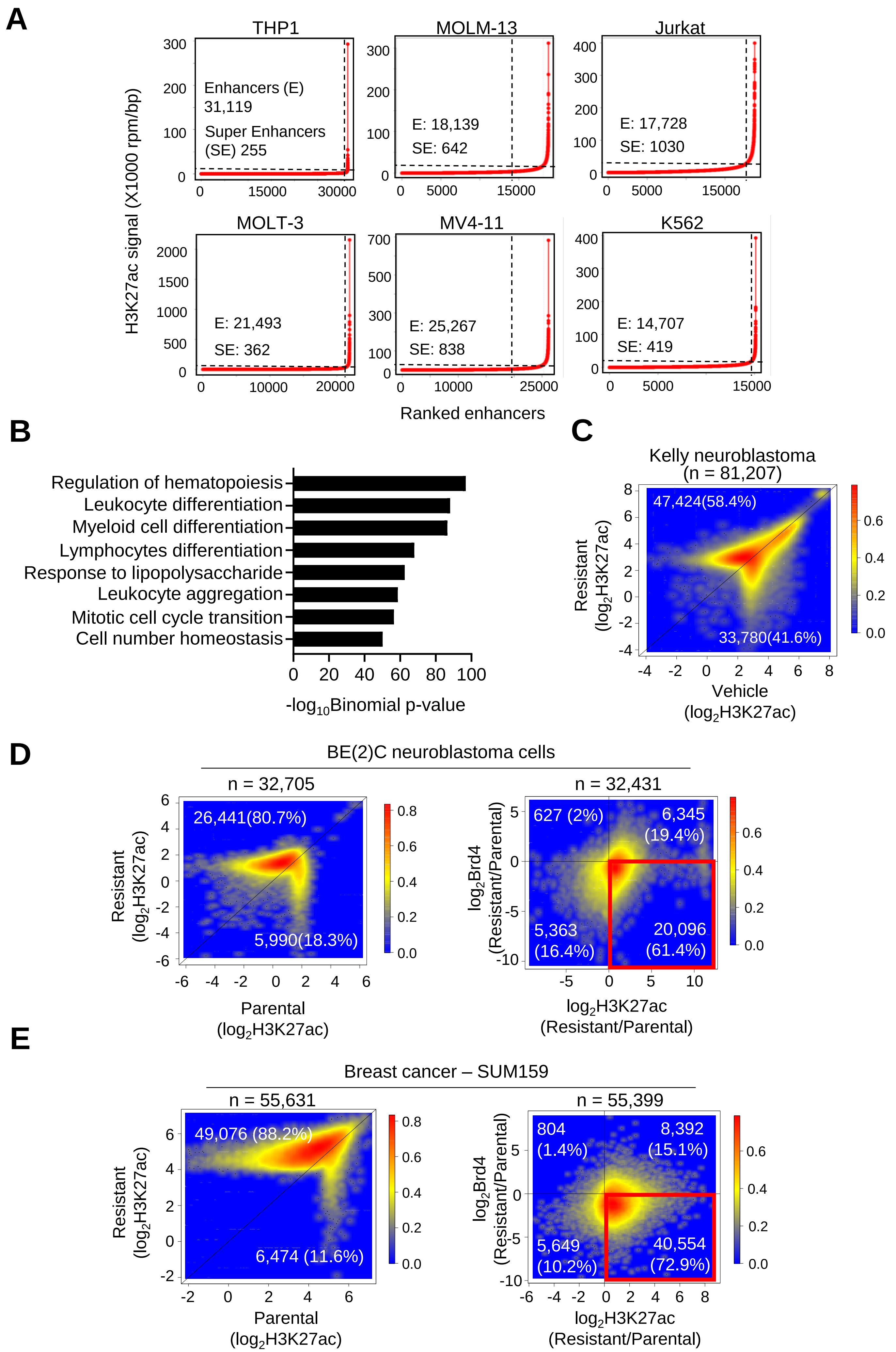

Supplementary Figure 1. Dynamic enhancer remodeling in multiple BETi-resistant cancer cells (related to Figure 1)

(A) Identification of super-enhancers based on H3K27ac enrichment in the indicated cell lines. The numbers of enhancers (E) and super-enhancers (SE) were listed.

(B) Genomic Regions Enrichment of Annotations Tool (GREAT) analysis of randomly selected genomic regions (n = 15,411) with gained H3K27ac regardless of BRD4 binding in BETi-resistant MLL-AF9 AML cells.

(C) Scatter plots of H3K27ac enriched regions in BETi-sensitive (parental, x-axis) and BETi-resistant (y-axis) Kelly neuroblastoma cells. The enhancer regions were identified based on the publicly available H3K27ac ChIP-seq data as listed in Table S1.

(D-E left) Scatter plot representation of differential H3K27ac-enriched regions in paired BETi-sensitive (parental, x-axis) and BETi-resistant (y-axis) BE(2)C neuroblastoma cells (D) or SUM159 breast cancer cells (E).

(D-E right) Scatter plot representation of differential H3K27ac and Brd4 enriched regions in paired BETi sensitive (parental, x-axis) and BETi resistant (y-axis) BE(2)C neuroblastoma cells (D) or SUM159 breast cancer cells (E). Red highlighted region: genomic regions displayed increased H3K27ac enrichment with no change or decreased BRD4 binding in BETi resistant BE(2)C neuroblastoma cells (D) or SUM159 (E) breast cancer cells.

Supplementary Figure 2

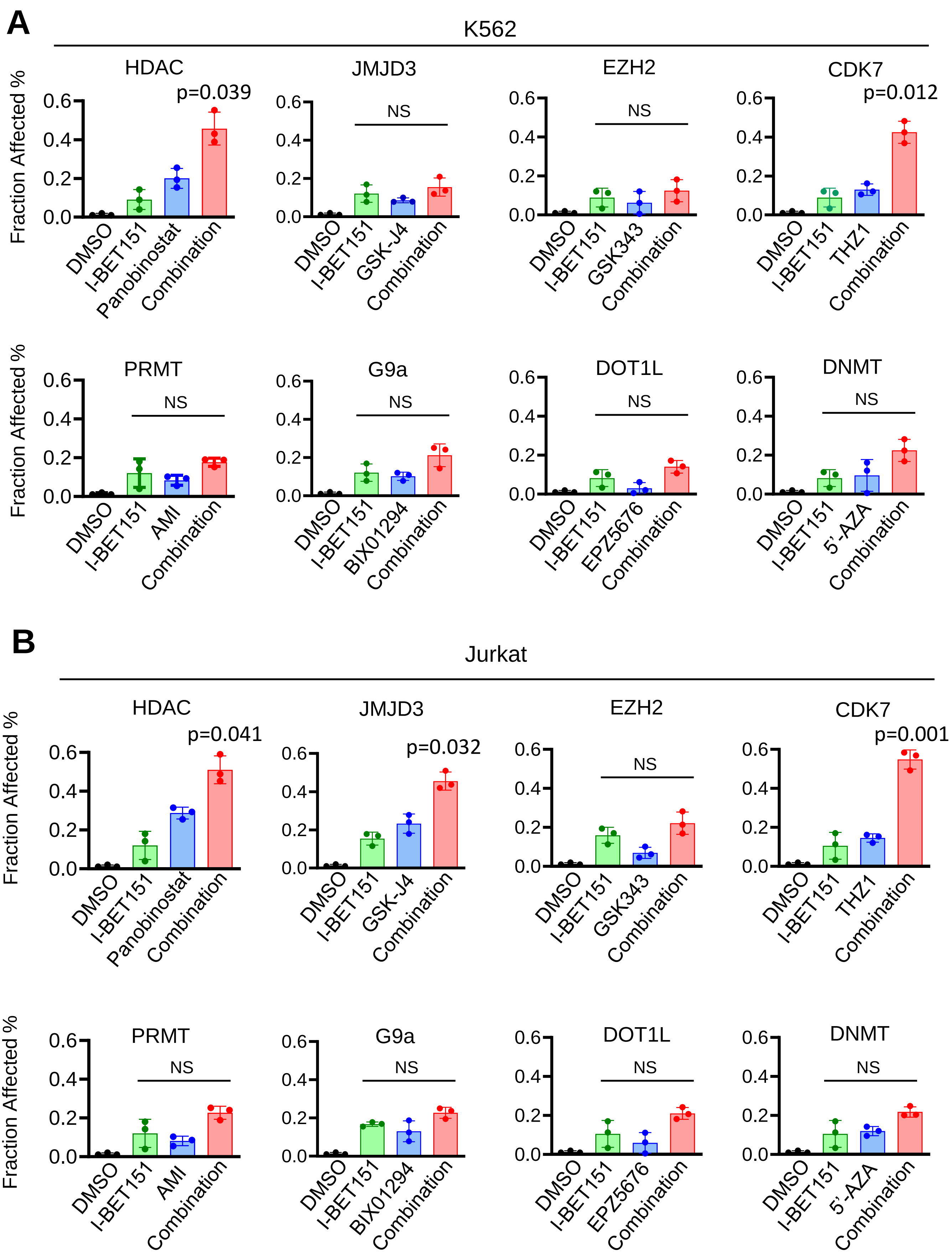

Supplementary Figure 2. Primary drug screening identifies the CDK7 inhibitor as a top candidate in overcoming BETi resistance (related to Figure 1)

(A-B) Cell viability of K562 cells (A) and Jurkat cells (B) after 72-hour treatment with I-BET151 (BETi, 2.5 μM) and other epigenetic inhibitors: GSK-J4 (JMJD3i, 2 μM), GSK343 (EZH2i, 1 μM), AMI (PRMTi, 2 μM), BIX01294 (G9ai, 2 μM), EPZ5676 (DOT1Li, 1 μM), 5'-AZA (DNMTi, 0.5 μM) and THZ1 (CDK7i, 10 nM). Data were shown as mean ± S.D; n = 3 from 3 independent experiments, NS: not significant, by two-tailed Student's t-test.

Supplementary Figure 3

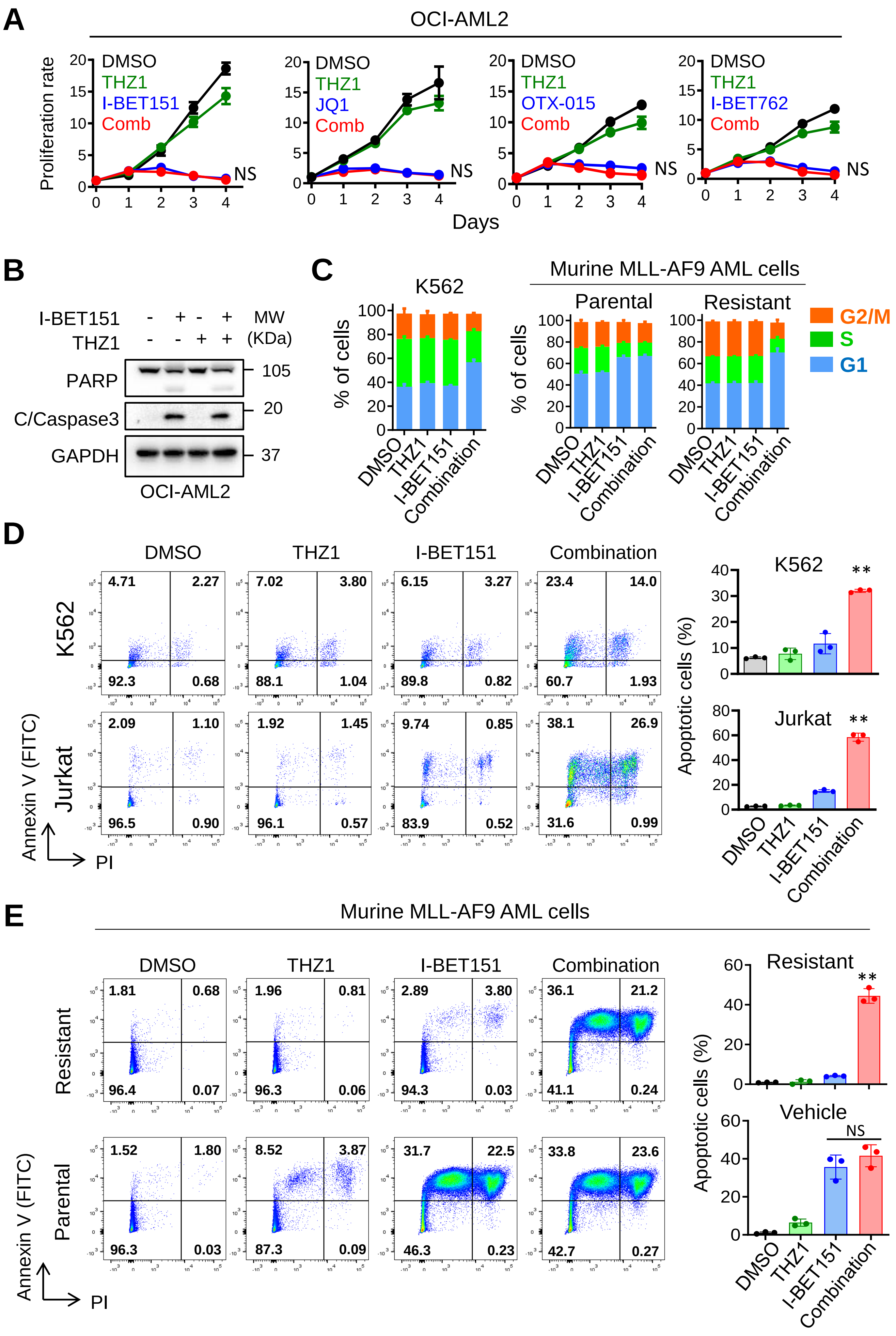

Supplementary Figure 3. THZ1 and I-BET151 synergistically induces cell cycle arrest and apoptosis in BETi-resistant leukemia cells. (Related to Figure 2)

(A) Proliferation analysis of OCI-AML2 cells treated with DMSO (black), THZ1 (green), BET inhibitors (blue), and combination of THZ1 and BET inhibitors (red). THZ1: 12.5 nM, I-BET151 and OTX-015: 2.5 μM, I-BET762: 5 μM. Data were shown as mean ± S.D; n = 6 from 3 independent experiments, NS: not significant, by two-tailed Student's t-test.

(B) Immunoblot analysis of the expression of apoptosis marker PARP and cleaved Caspase3 (C/Caspase3) in OCI-AML2 cells treated with DMSO, THZ1, I-BET151, and the combination of THZ1 + I-BET151 for 24 hours (n = 3 from 3 independent experiments). GAPDH was used as loading control. The same inhibitor concentrations were used as in Figure S3A.

(C) Cell cycle analysis of K562 and murine AF9 AML cells after DMSO, THZ1 (12.5 nM), I-BET151 (2.5 μM), and the combination treatment for 24 hours. Data were shown as mean ± S.D; n = 3 from 3 independent experiments.

(D-E) Representative flow cytometry analysis (left) and quantification (right) of K562, Jurkat (D) and murine AF9 AML (E) cell death analysis measured by Annexin V and propidium iodide (PI) staining 24 hrs after DMSO, THZ1, I-BET151, and the combination treatment. Data were shown as mean ± S.D; n = 3 from 3 independent experiments, ** p =0.00022 (K562), 0.00123 (Jurkat) and 0.00226 (AF9 resistant); NS: not significant, by two-tailed Student's t-test.

Supplementary Figure 4

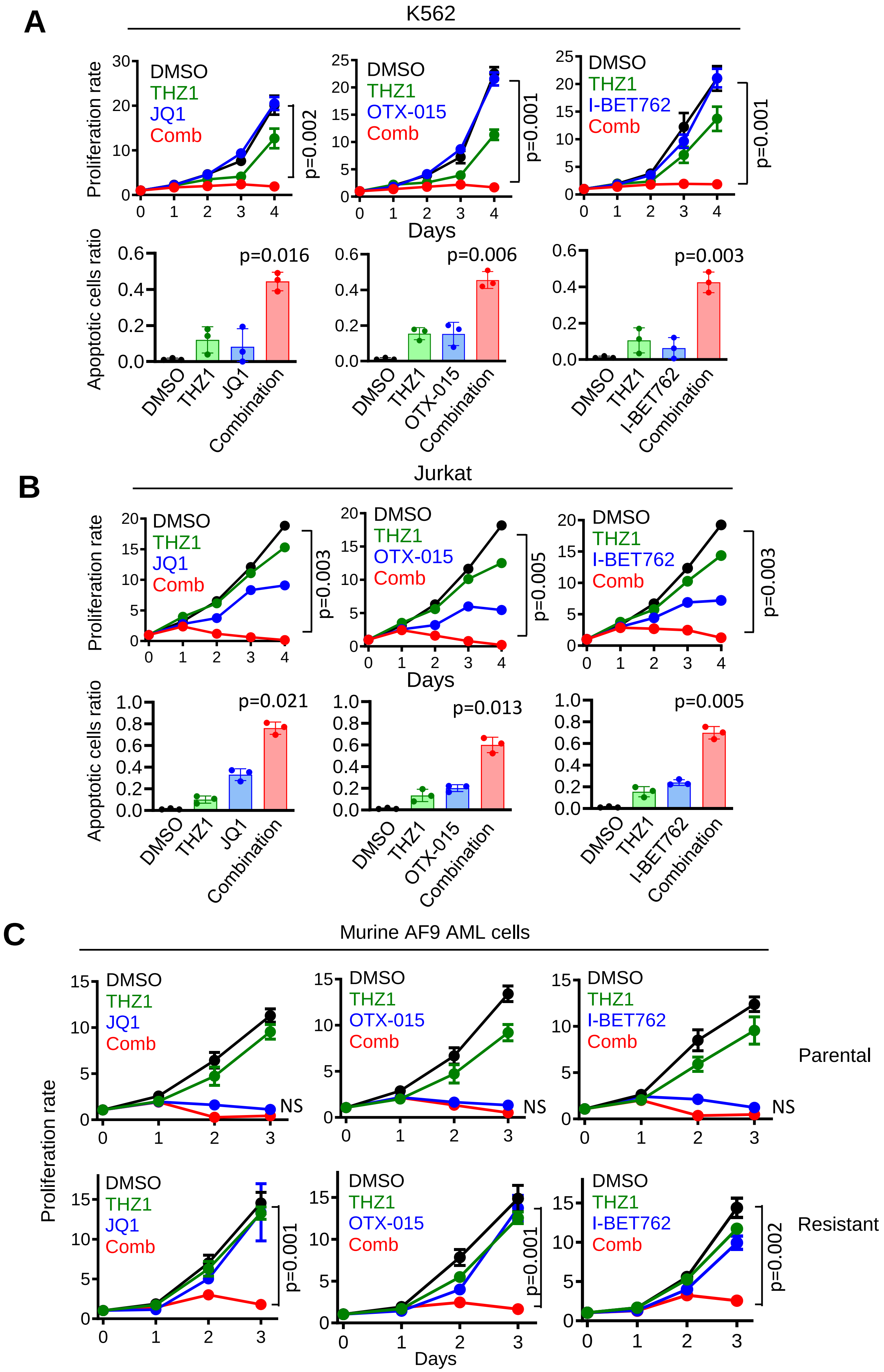

Supplementary Figure 4. Cross-synergy of a CDK7 inhibitor and various BET inhibitors in BETi-resistant cells. (Related to Figure 2)

(A-B) Proliferation and apoptosis analysis of K562 (A) and Jurkat (B) cells treated with the indicated inhibitors. The concentrations of JQ1, OTX-015, I-BET762 and THZ1 are 2.5 μM, 2.5 μM, 5 μM and12.5 nM, respectively for K562; 0.625 μM, 1.25 μM, 2.5 μM and 3.125 nM, respectively for Jurkat cells. Data were shown as mean ± S.D; n = 6 from 3 independent experiments by two-tailed Student's t-test.

(C) Proliferation analysis of murine AF9 AML cells treated with different BET inhibitors and THZ1. The concentrations of JQ1, OTX-015, I-BET762 and THZ1 are 0.325 μM, 0.625 μM, 1.25 μM and 50 nM, respectively. Data were shown as mean ± S.D; n = 6 from 3 independent experiments, by two-tailed Student's t-test.

Supplementary Figure 5

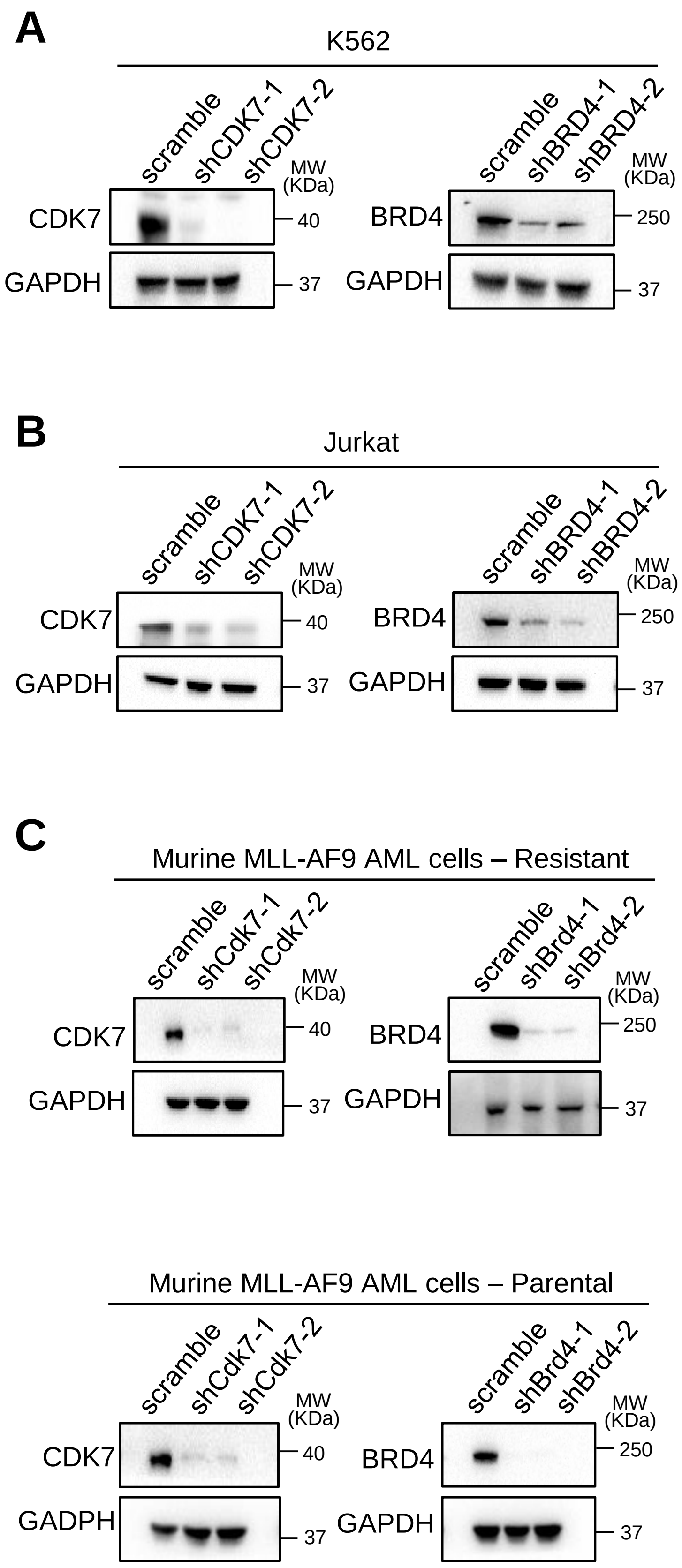

Supplementary Figure 5. Validation of BRD4 and CDK7 knock-down efficiency. (Related to Figure 2)

(A-C) Two independent shRNAs were transduced into K562 (A), Jurkat (B) and murine AF9 AML cells (C), and then selected with puromycin (3 μM) for 3 days. Positive living cells were harvest and subjected to Western blotting with BRD4 and CDK7 antibodies. GAPDH was used as loading control. 3 independent experiments were performed.

Supplementary Figure 6

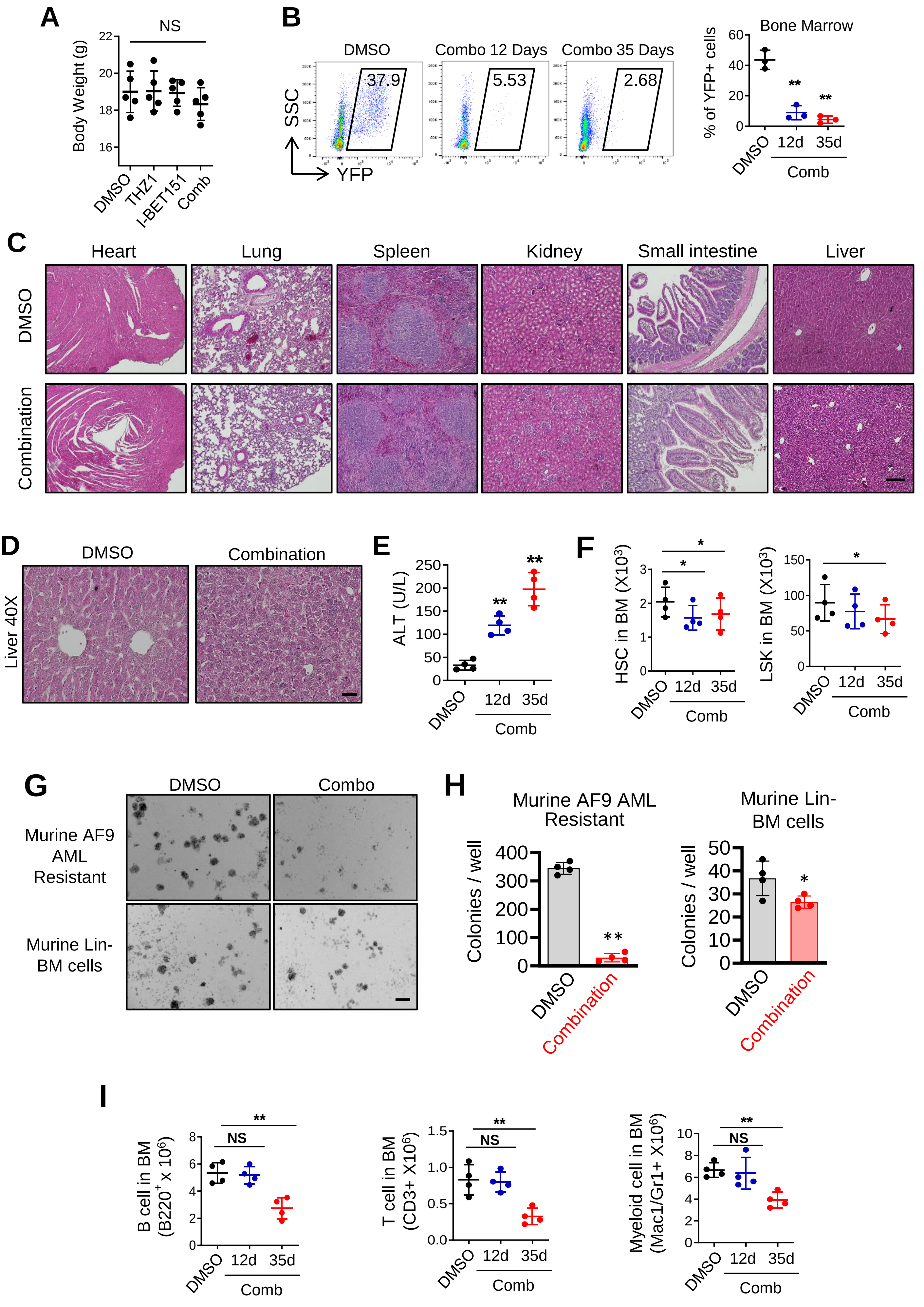

Supplementary Figure 6. Synthetic lethality of dual inhibition of CDK7 and BRD4 in BETi-resistant leukemia cells in vivo. (Related to Figure 3)

(A) Statistical analysis of body weight of recipient mice transferred with BETi-resistant murine AF9 AML cells at 20 days after DMSO, THZ1, I-BET151, or the combination treatment with THZ1 and I-BET151. Data were shown as mean ± S.D; n = 5 mice. NS: Not significant, by one-way ANOVA with Dunnett's post-hoc correction.

(B) Representative flow cytometry analysis (left) and quantification (right) of YFP-positive leukemic cells in bone marrow (BM) of mice treated with DMSO (black) or the combination of THZ1 and I-BET151 for 2 weeks (blue) or 5 weeks (red). Data were shown as mean ± S.D; n = 3 independent mice. ** p = 0.0028 (12d) and 0.0056 (35d), by two-tailed Student's t-test.

(C) Representative HE stained sections of heart, lung, spleen, kidney, small intestine and liver collected from the normal mice treated DMSO or combination (THZ1 and I-BET151, bottom) (n = 3 independent mice). Scale bar: 200 μm.

(D) Zoomed-in images (40 x) of liver section from the normal mice treated with DMSO (left) or receiving the combination therapy (THZ1 and I-BET151, right) (n = 3). Scale bar: 25 μm.

(E) Serum ALT levels in mice treated with DMSO (black) or the combination of THZ1 and I-BET151 for 2 weeks (blue) or 5 weeks (red). Data were shown as mean ± S.D; n = 4 mice. ** p = 0.0019 (12d) and 0.0047 (35d), by two-tailed Student's t-test.

(F) Absolute numbers of indicated cells isolated from bone marrow of mice treated with DMSO (black) or the combination of THZ1 and I-BET151 for 2 weeks (blue) or 5 weeks (red). Data were shown as mean ± S.D; n = 4 mice. NS: Not significant, ** p < 0.005, * p < 0.01 by two-tailed Student's t-test.

(G-H) Representative images (G) and quantification (H) of colony-forming unit (CFU) assay results using primary BM Lin- cells. BETi-resistant murine MLL-AF9 AML cells were used as control. Data were shown as mean ± S.D; n = 4 from 4 independent experiments, ** p = 0.0001, * p = 0.04 by two-tailed Student's t-test. Scale bar, 200 μm.

(I) Absolute numbers of the indicated cells isolated from bone marrow of mice treated with DMSO (black) or the combination of THZ1 and I-BET151 for 2 weeks (blue) or 5 weeks (red). Data were shown as mean ± S.D; n = 4 mice. NS, Not significant; ** p=0.01 (B cell), 0.002 (T cell) and 0.005 (Myeloid cell) by two-tailed Student's t-test.

Supplementary Figure 7

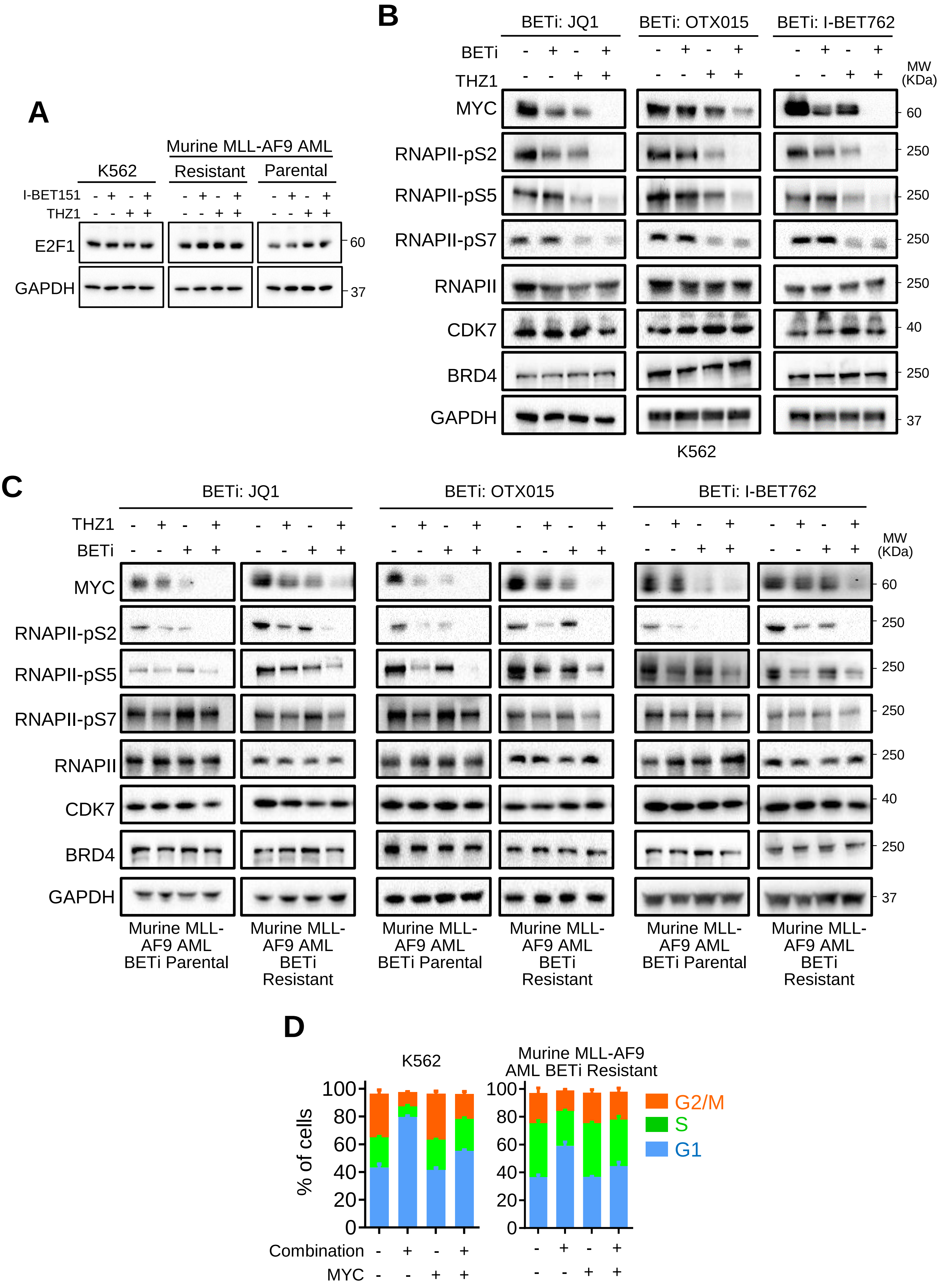

Supplementary Figure 7. THZ1 synergizes with BET inhibitors with varying chemical structures to reduce MYC expression. (Related to Figure 4).

(A) Immunoblot analysis of the protein levels of E2F1 in K562 cells and murine AF9 AML cells after DMSO, I-BET151, THZ1, and the combination treatment for 24 hrs. GAPDH was used as control (3 independent assays were performed).

(B-C) Representative Western blotting showing the protein levels of MYC, CDK7, BRD4, phosphorylated and total RNAPII in K562 cells (A) and murine AF9 AML cells (B) after DMSO, I-BET151, THZ1, and combination treatment for 24 hrs. GAPDH was used as control. 3 independent assays were performed.

(D) Cell cycle analysis measured by PI staining of K562 and BETi-resistant murine AF9 AML cells expressing the empty vector (EV) or MYC treated with DMSO or the combination therapy (Combo) for 24 hours. Data were shown as mean ± S.D; n = 3 from 3 independent experiments. Same drug doses were used as in Figure 4.

Supplementary Figure 8

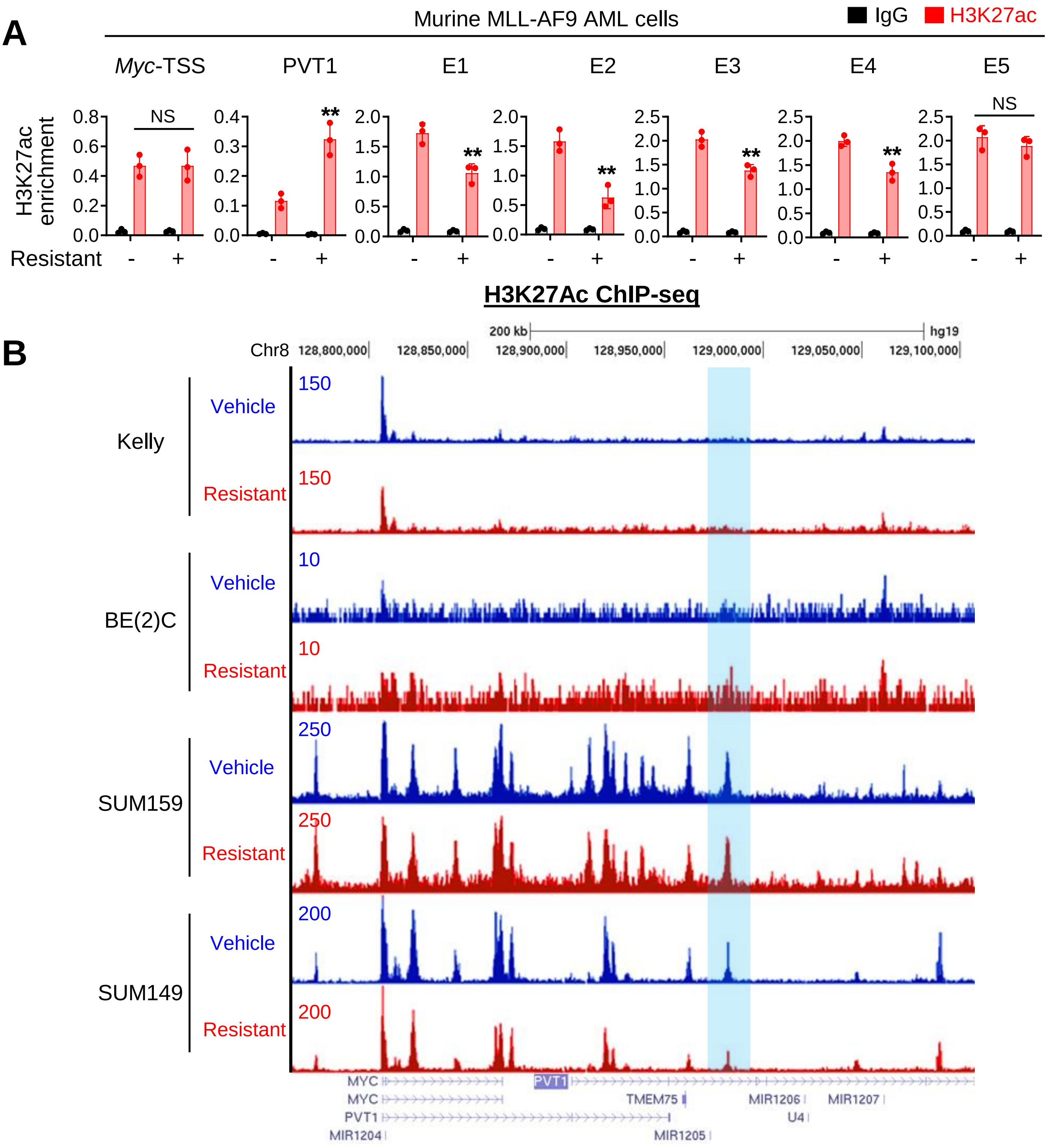

Supplementary Figure 8. Leukemia-specific gain of H3K27ac at the PVT1 locus in BETi-resistant tumor cells. (Related to Figure 5)

(A) ChIP-qPCR analysis of H3K27ac enrichment at Myc transcription starting site (TSS), Pvt1 and BENC enhancers in BETi-sensitive or resistant murine AF9 AML cells. Data were shown as mean ± S.D; n = 3 from 3 independent experiments, NS: Not significant ; ** p = 0.0451 (PVT1), 0.0027 (E1), 0.0236 (E2), 0.0382 (E3) and 0.0487 (E4), by two-tailed Student's t-test.

(B) Genome browser views of H3K27ac enrichment at the MYC PVT1 enhancers in parental (vehicle, blue) and the corresponding resistant cells (red) among different types of cancer cells. H3K27ac ChIP-seq data were obtained from publicly available database listed in Supplementary Data2.

Supplementary Figure 9

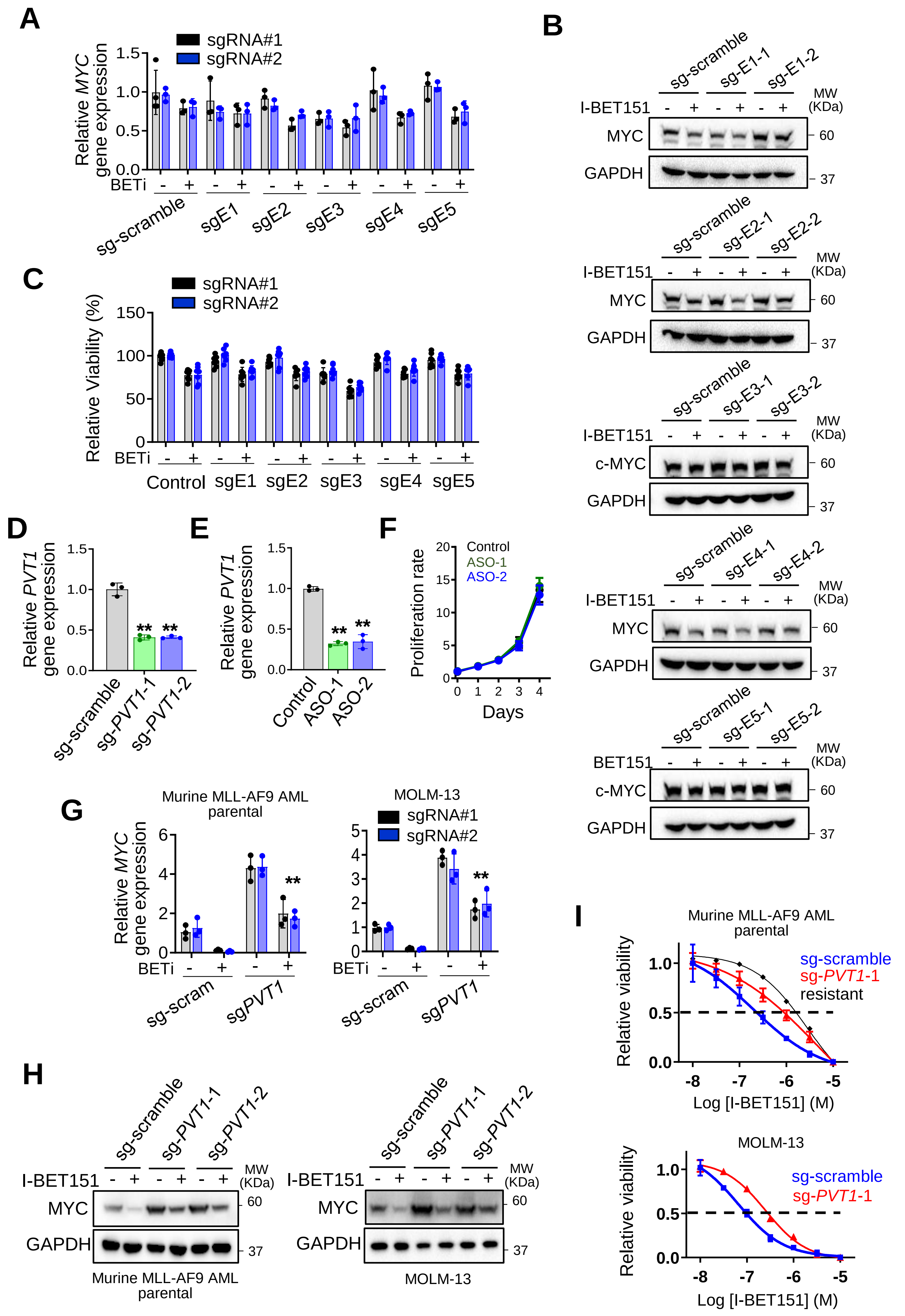

Supplementary Figure 9. PVT1 enhancer, but not BENC is indispensable for Myc expression in BETi-resistant leukemia cells. (Related to Figure 6)

(A-C): mRNA (B), protein (C) levels and cell viability (D) of K562 cells expressing dCas9-KRAB and two independent sgRNAs targeted to BENC after I-BET151 (2.5 μM) treatment for 36 hrs. K562 cells with dCas9-KRAB and a scrambled sgRNA were used as control. Data were shown as mean ± S.D; n=3 from 3 independent experiments.

(D) Real-time quantitative PCR (qPCR) analysis of PVT1 expression in K562 cells transduced with a scrambled sgRNA or sgRNA targeted to the PVT1 locus (n = 3 from 3 independent experiments). ** p = 0.0021(sgPVT1-1) and 0.0056 (sgPVT1-2), by two-tailed Student's t-test.

(E) Real-time qPCR analysis of PVT1 expression in K562 cells treated with PVT1-targeting ASOs. ** p = 0.002 (ASO-1) and 0.0037 (ASO-2), by two-tailed Student's t-test (n = 3 from 3 independent experiments).

(F) Growth curves of K562 cells in the absence (control) or presence of PVT1-targeting ASO treatment (Data were shown as mean ± S.D; n = 6 from 3 independent experiments).

(G-I) Gene transcription (G), protein expression levels (H) and dose response curves (I) of parental murine MLL-AF9 AML cells and MOLM-13 cells expressing dCas9-p300Core and two independent sgRNAs targeted to PVT1 after BETi treatment. Data were shown as mean ± S.D; n = 3(G) and 8(I) from 3 independent experiments, ** p = 0.0004 (AF9) and 0.0002 (MOLM-13), by two-tailed Student's t-test.
