# Supplementary material for: A combination strategy targeting enhancer plasticity exerts synergistic lethality against BETi-resistant leukemia cells
Source: Nat Commun. 2020 Feb 6;11:740. doi: 10.1038/s41467-020-14604-6 (PMC7005144; doi:10.1038/s41467-020-14604-6)
Supplement: Supplementary file 3 — Description of Additional Supplementary Files [file 41467_2020_14604_MOESM3_ESM.pdf]

Description of Additional Supplementary Files

File Name: Supplementary Data 1

Description: Full lists of public data accession numbers

File Name: Supplementary Data 2

Description: Original data for the complete blood count (CBC) assay.

File Name: Supplementary Data 3

Description: Antibody information and primer sequences.
