# Supplementary material for: A combination strategy targeting enhancer plasticity exerts synergistic lethality against BETi-resistant leukemia cells
Source: Nat Commun. 2020 Feb 6;11:740. doi: 10.1038/s41467-020-14604-6 (PMC7005144; doi:10.1038/s41467-020-14604-6)
Supplement: Supplementary file 7 — Reporting Summary [file 41467_2020_14604_MOESM7_ESM.pdf]

nature research

Corresponding author(s): Yun Huang

Last updated by author(s): Dec 30, 2019

Reporting Summary

Nature Research wishes to improve the reproducibility of the work that we publish. This form provides structure for consistency and transparency in reporting. For further information on Nature Research policies, see Authors & Referees and the Editorial Policy Checklist.

Statistics

For all statistical analyses, confirm that the following items are present in the figure legend, table legend, main text, or Methods section.

n/a	Confirmed	
☐	☒	The exact sample size (*n*) for each experimental group/condition, given as a discrete number and unit of measurement
☐	☒	A statement on whether measurements were taken from distinct samples or whether the same sample was measured repeatedly
☐	☒	The statistical test(s) used AND whether they are one- or two-sided<br>*Only common tests should be described solely by name; describe more complex techniques in the Methods section.*
☒	☐	A description of all covariates tested
☒	☐	A description of any assumptions or corrections, such as tests of normality and adjustment for multiple comparisons
☐	☒	A full description of the statistical parameters including central tendency (e.g. means) or other basic estimates (e.g. regression coefficient) AND variation (e.g. standard deviation) or associated estimates of uncertainty (e.g. confidence intervals)
☐	☒	For null hypothesis testing, the test statistic (e.g. *F*, *t*, *r*) with confidence intervals, effect sizes, degrees of freedom and *P* value noted<br>*Give P values as exact values whenever suitable.*
☒	☐	For Bayesian analysis, information on the choice of priors and Markov chain Monte Carlo settings
☒	☐	For hierarchical and complex designs, identification of the appropriate level for tests and full reporting of outcomes
☒	☐	Estimates of effect sizes (e.g. Cohen's *d*, Pearson's *r*), indicating how they were calculated

Our web collection on statistics for biologists contains articles on many of the points above.

Software and code

Policy information about availability of computer code

Data collection	NexSeq500 (illumina)<br>Eclipse Ci microscopy (Nikon)<br>ChemiDoc imaging system (Bio-Rad)<br>LightCycler 96 (Roche)

Data analysis	Image/Western blot analysis: Fiji (Image J) (Version 1.51n); Image Lab (BioRad; Version 6.0).<br><br>Statistical analysis: GraphPad Prism8.0<br><br>RNA-seq data were mapped to hg19 or mm10 genome assembly using tophat-2.1.1 with default parameters. DESeq2 with default parameters were used to call significantly differentially expressed genes (DEGs) (q-value <= 0.05) among DMSO, THZ1, I-BET151 and combination groups. The merged DEGs among the four groups were used to perform Principal Component Analysis (PCA). Heatmaps for gene expression were plotted using the heatmap3 R package. DEGs functional enrichment was performed using GSEA. All the ChIP-seq data were mapped to hg19 or mm10 genome assembly using bowtie2 with default parameters.<br><br>Only uniquely mapped reads and non-duplicated reads were used as input for MACS2 (default parameters) to call ChIP-seq peaks. SmoothScatter R packages was used to plot the density scatter plot for ChIP-seq data.<br>Super enhancers were identified using the ROSE pipeline (http://younglab.wi.mit.edu/super_enhancer_code.html).<br><br>Basic4C R package (Version 1.22.0) was used to calculate normalized 4C signals and plot 4C signals around Myc location.<br><br>Juicebox (Version 1.9.8) was used to perform visualization of HiC data and HiCHIP data.<br><br>Genomic Regions Enrichment of Annotations Tool (GREAT) (Version 3.0.0)

For manuscripts utilizing custom algorithms or software that are central to the research but not yet described in published literature, software must be made available to editors/reviewers. We strongly encourage code deposition in a community repository (e.g. GitHub). See the Nature Research guidelines for submitting code & software for further information.

Data

Policy information about availability of data

All manuscripts must include a data availability statement. This statement should provide the following information, where applicable:

- Accession codes, unique identifiers, or web links for publicly available datasets
- A list of figures that have associated raw data
- A description of any restrictions on data availability

H3K27ac ChIP-seq:
GSM733656
GSM1697882
GSM2136938
GSM2136946
GSM2108046
GSM1519644
GSM2876322
GSM2876323
GSM2876322
GSM2876323
GSM1553142
GSM1553137
GSM1897161
GSM1897159
GSM3231641
GSM3231642
GSM1842713
GSM1842708

Brd4 ChIP-seq:
GSM3231633
GSM3231634
GSM1842711
GSM1842706
GSM1897160
GSM1897158

ATAC-seq:
GSM1782764
GSM2544216

Hi-C:
GSE63525
PRJNA385337

H3K27ac HiChIP:
GSE101498

RNAPII ChIP-seq:
GSM2635250

HDAC1 ChIP-seq:
GSE91720

CEBPB ChIP-seq:
GSE91748

RUNX1 ChIP-seq:
GSE96253

GATA1 ChIP-seq:
GSM1782703

GATA2 ChIP-seq:
GSM777641

EZH2 ChIP-seq:
GSM830992

JARID1C ChIP-seq:
GSM831000

LSD1 ChIP-seq:
GSM831002

P300 ChIP-seq:
GSM831006

REST ChIP-seq:
GSM831015

SIRT6 ChIP-seq:
GSM831020

SUZ12 ChIP-seq:
GSM831021

TAL1 ChIP-seq
GSM935496

RNA-Seq:
PRJNA543382

Field-specific reporting

Please select the one below that is the best fit for your research. If you are not sure, read the appropriate sections before making your selection.

☒ Life sciences ☐ Behavioural & social sciences ☐ Ecological, evolutionary & environmental sciences

For a reference copy of the document with all sections, see nature.com/documents/nr-reporting-summary-flat.pdf

Life sciences study design

All studies must disclose on these points even when the disclosure is negative.

Sample size	Sample size was decided by our experience with the techniques in this research. From our statistic results, we were confident that the sample size we used was sufficient to support our results.
Data exclusions	None
Replication	Biological replicates (>=2) were performed for bulk RNA-seq and 4C-seq studies. We also performed more than 3 biological replicates for the western blot, dot blot and qPCR experiments by collecting materials from independent samples. The replicate number was reported in the figure legends. All replications were successful.
Randomization	BETi resistant MLL-AF9-YFP murine leukemia cells (1 million) were injected intravenously into B6.SJL-Ptprca Pepcb/BoyJ mice (CD45.1, 6-8 weeks old; female) after sub-lethal irradiation (300 cGy). Treatment with vehicle, I-BET151, THZ1 or the combination regime (I-BET151 + THZ1) commenced after engraftment of leukemia as determined by the presence of >1% yellow fluorescent protein (YFP) in the peripheral blood of recipient mice. Mice were randomly assigned into one of the four above-mentioned treatment groups (5 mice/group).
Blinding	Investigators were not blinded in this study because no clinical relevant experiments were performed. However, there is no bias for all the data collected in this study.

Reporting for specific materials, systems and methods

We require information from authors about some types of materials, experimental systems and methods used in many studies. Here, indicate whether each material, system or method listed is relevant to your study. If you are not sure if a list item applies to your research, read the appropriate section before selecting a response.

Materials & experimental systems

n/a	Involved in the study	
☐	☒	Antibodies
☐	☒	Eukaryotic cell lines
☒	☐	Palaeontology
☐	☒	Animals and other organisms
☒	☐	Human research participants
☒	☐	Clinical data

Methods

n/a	Involved in the study	
☒	☐	ChIP-seq
☐	☒	Flow cytometry
☒	☐	MRI-based neuroimaging

Antibodies

Antibodies used

PARP Cell Signaling 9542 1:1000
cleaved caspase3 Cell Signaling 9664 1:1000
c-myc Abcam ab32072 1:1000
Brd4 Bethyl A301-985A 1:500
Cdk7 Bethyl A300-705A 1:500
Pol 2 Bethyl A300-653A 1:1000
Pol 2 pS2 Bethyl A300-654A 1:500
Pol 2 pS5 Bethyl A300-408A 1:500
Pol 2 pS7 Millipore 04-1570 1:500
GAPDH Sigma G9545 1:3000
H3K27ac Abcam ab4729 1:100
H3K9me3 Abcam ab8898 1:100
Mac-1 Thermo fisher (M1/70) 1:200
Gr-1 Thermo fisher (RB6-8C5) 1:200
CD4 Thermo fisher GK1.5 1:200
B220 Thermo fisher (RA3-6B2) 1:200
CD19 Thermo fisher eBio1D3 1:200
Ter119 Thermo fisher (Ter119) 1:50
CD71 Thermo fisher RI7217 1:50
c-Kit APC Thermo fisher (2B8) 1:400
Sca-1 PE/Cy7 Thermo fisher (D7) 1:400
CD150 PE Thermo fisher (TC15-12F12.2) 1:100
CD48 FITC Thermo fisher (HM48-1) 1:100
CD16/32-PE Thermo fisher 93 1:300
CD34 FITC Thermo fisher (RAM34) 1:100

Validation	PARP, cleaved caspase3, c-myc, brd4, cdk7, Pol2 antibodies were validated by Western Blot, H3K27ac and H3K9me3 antibodies were validated by ChIP-seq. The rest of antibodies were validated by their respective companies.

Eukaryotic cell lines

Policy information about cell lines

Cell line source(s)	K562, Jurkat, OCI-2, 293T cells were obtained from ATCC, and murine MLL-AF9 leukemia cells were gifts from Dr. Mark A Dawson (Peter MacCallum Cancer Centre, Melbourne, Victoria, Australia)
Authentication	K562, Jurkat, OCI-2, 293T cells were authenticated by ATCC, and murine MLL-AF9 leukemia cells were authenticated by Dr. Mark A Dawson's group (Peter MacCallum Cancer Centre, Melbourne, Victoria, Australia).
Mycoplasma contamination	cells were determined to be mycoplasma negative
Commonly misidentified lines (See ICLAC register)	none

Animals and other organisms

Policy information about studies involving animals; ARRIVE guidelines recommended for reporting animal research

Laboratory animals	B6.SJL-Ptprca Pepcb/BoyJ mice (CD45.1, 6-8 weeks old; female)
Wild animals	not involved in this study
Field-collected samples	not involved in this study
Ethics oversight	Animal studies were approved by the Institutional Animal Care Use Committee (IACUC) of the Institute of Biosciences and Technology, Texas A&M University.

Note that full information on the approval of the study protocol must also be provided in the manuscript.

Flow Cytometry

Plots

Confirm that:

☒ The axis labels state the marker and fluorochrome used (e.g. CD4-FITC).

☒ The axis scales are clearly visible. Include numbers along axes only for bottom left plot of group (a 'group' is an analysis of identical markers).

☒ All plots are contour plots with outliers or pseudocolor plots.

☒ A numerical value for number of cells or percentage (with statistics) is provided.

Methodology

Sample preparation	Bone marrow was harvested by flushing two femurs and two tibias. Cells were re-suspended in FACS buffer (PBS with 0.1% BSA, 2mM EDTA) and incubated with indicated dyes for 30 minutes at 4°C in the dark, followed by washing with FACS buffer twice.
Instrument	Flow cytometry analysis was performed using LSRII (BD Biosciences).
Software	Data were analyzed with the FlowJo10 software package (FlowJo,LLC).
Cell population abundance	Annexin V and PI positive cells were less than 5% in untreated cells. After drug treatments, the population varied from 10%-60%. YFP positive cell populations were around 40% in untreated mice and dropped to less than 10% in combination treatment. Exact numbers were displayed in Supplementary Figure 3D-E and Supplementary Figure 6B.
Gating strategy	Figures exemplifying the gating strategy were provided in the Supplementary Information (Supplementary Figure 3D-E, Supplementary Figure 6B). Singlets were gated on FSC-A/FSC-W as well as SSC-A/SSC-W

☒ Tick this box to confirm that a figure exemplifying the gating strategy is provided in the Supplementary Information.
