# Supplementary material for: A combination strategy targeting enhancer plasticity exerts synergistic lethality against BETi-resistant leukemia cells
Source: Nat Commun. 2020 Feb 6;11:740. doi: 10.1038/s41467-020-14604-6 (PMC7005144; doi:10.1038/s41467-020-14604-6)

Figure 2D

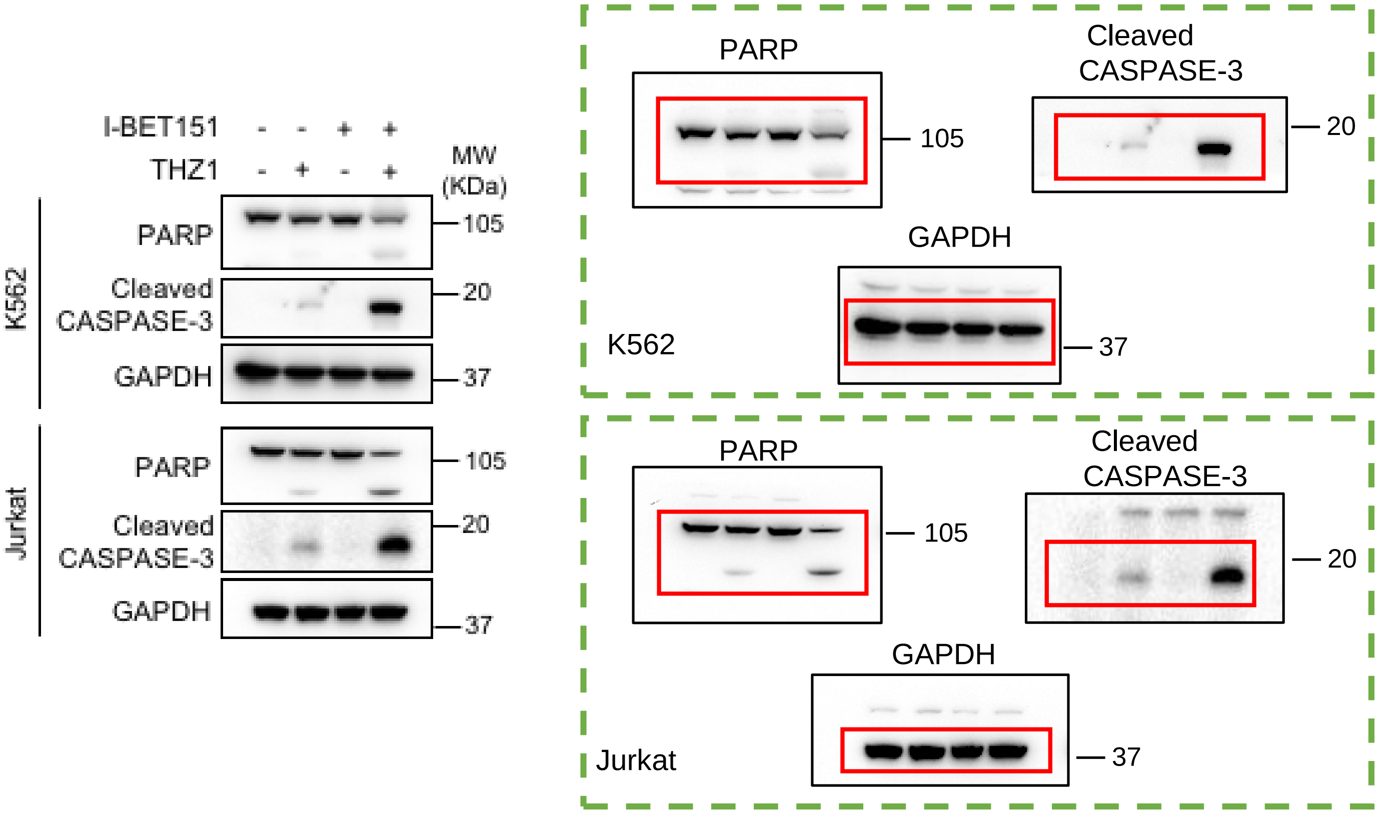

Figure 2E

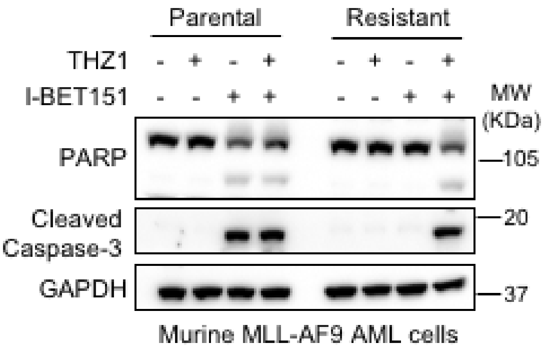

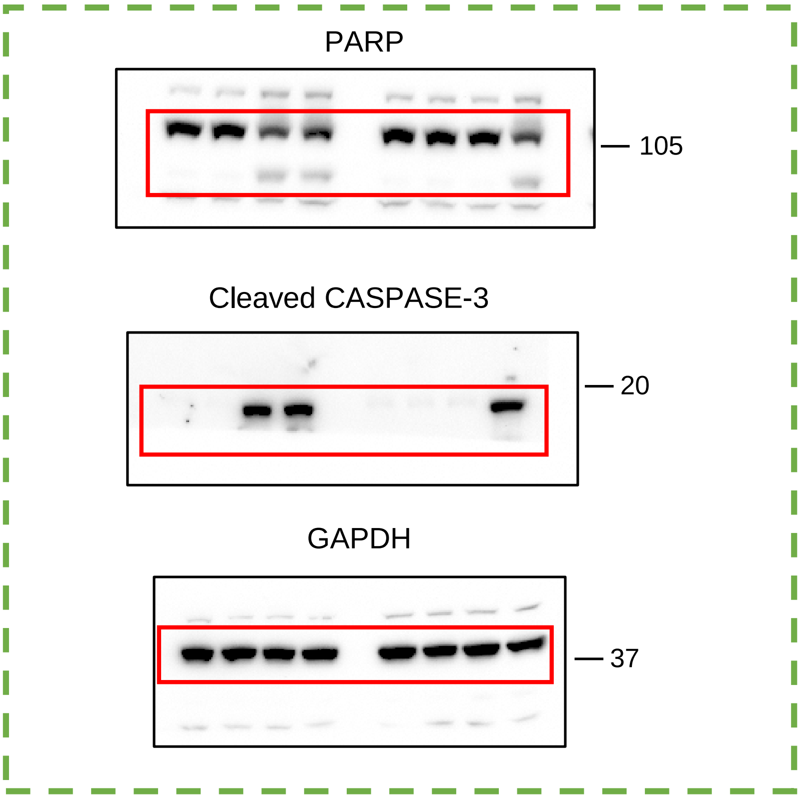

Figure 4F

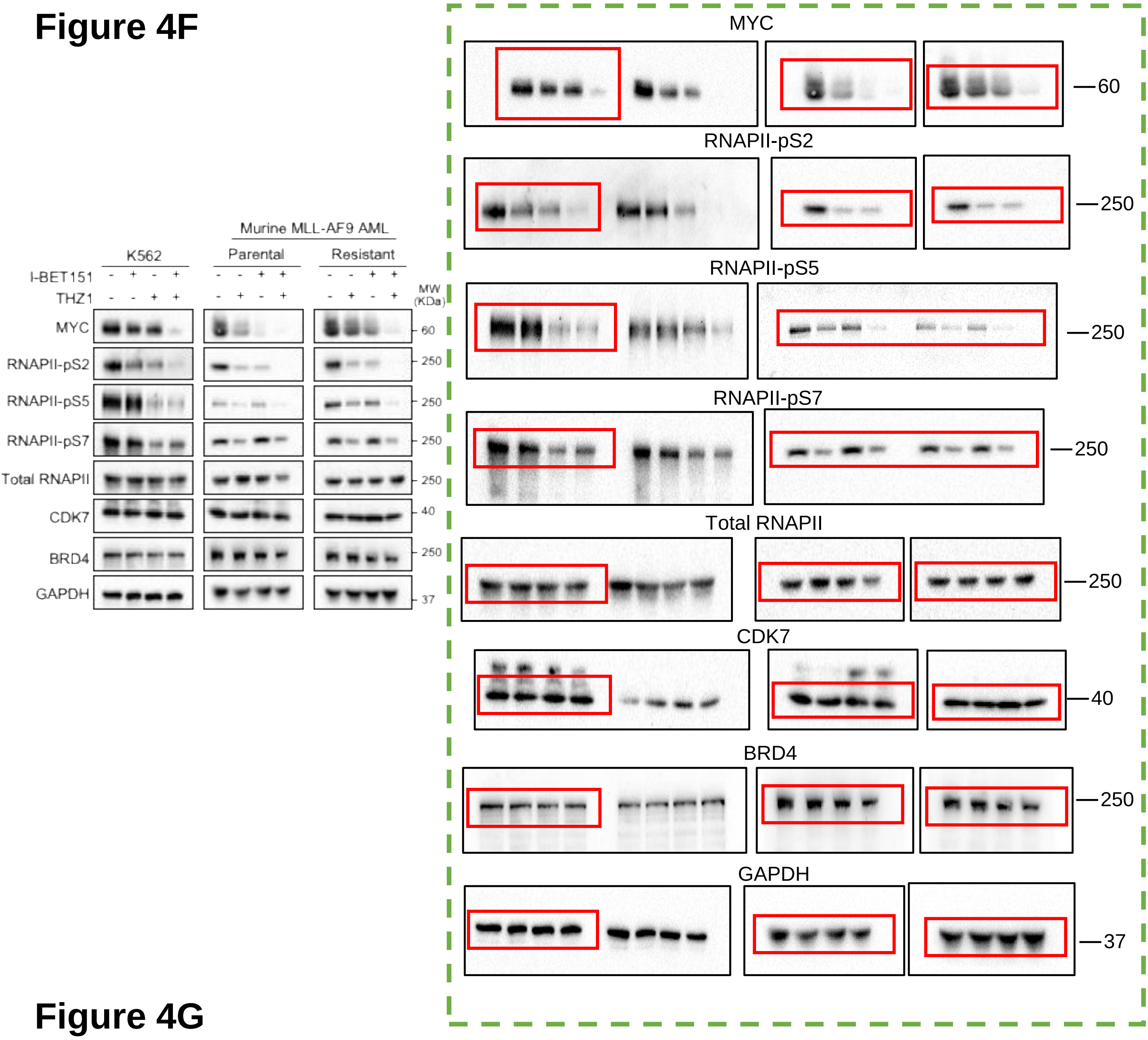

Figure 4G

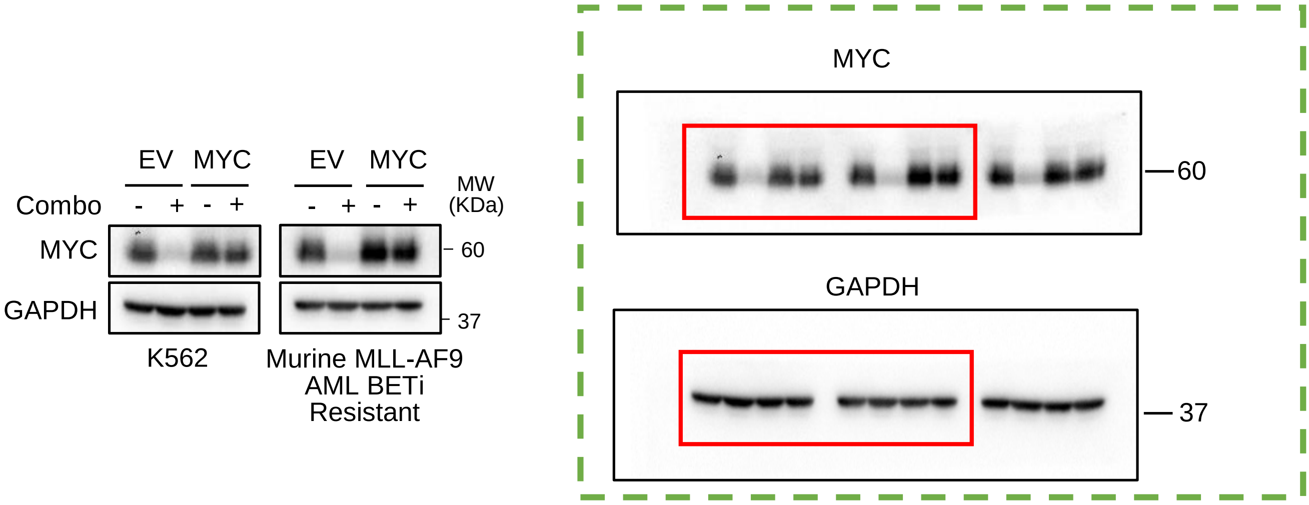

Figure 6D

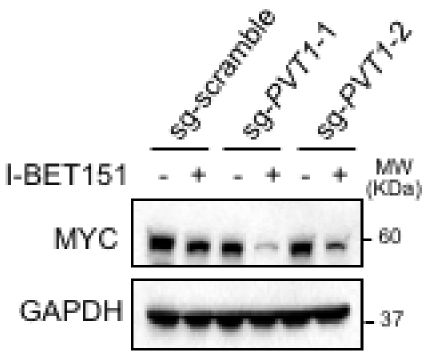

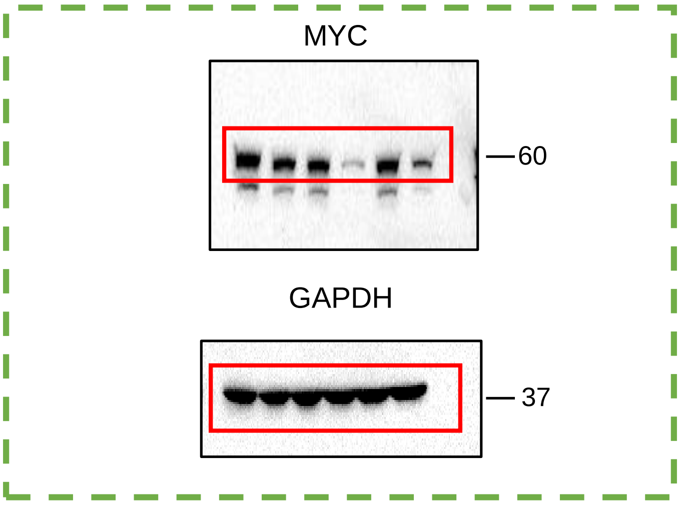

Figure 6I

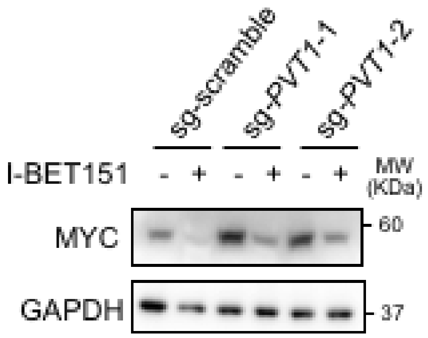

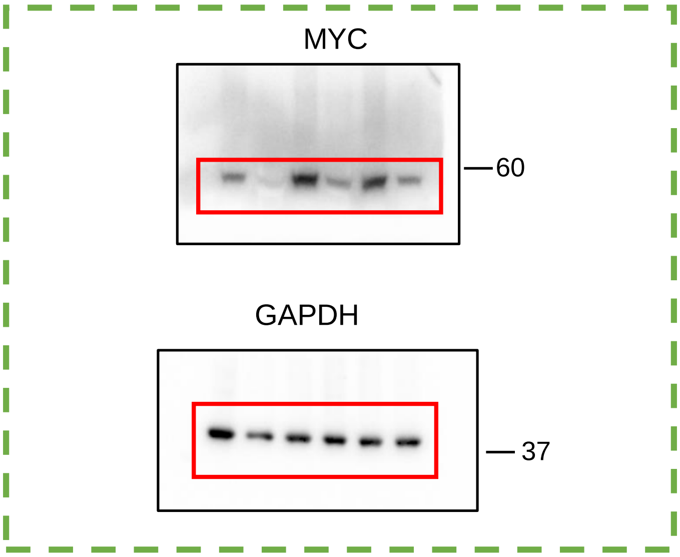

Figure S3B

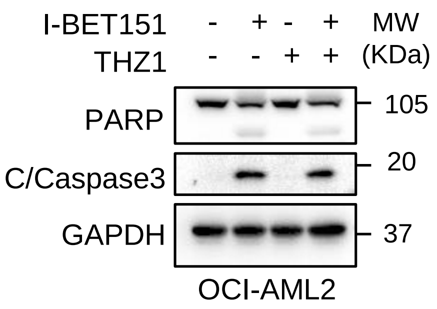
I-BET151 - + - +
THZ1 - - + +
MW
(KDa)
PARP
105
C/Caspase3
20
GAPDH
37
OCI-AML2

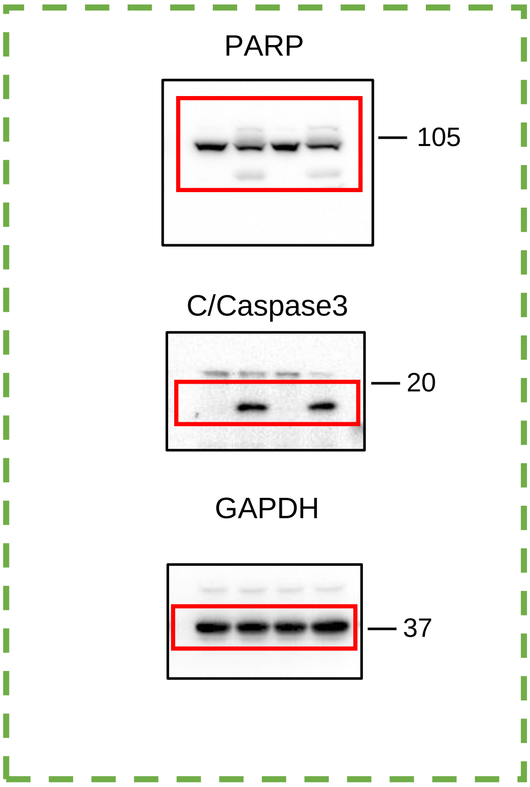
PARP
105
C/Caspase3
20
GAPDH
37

Figure S5A

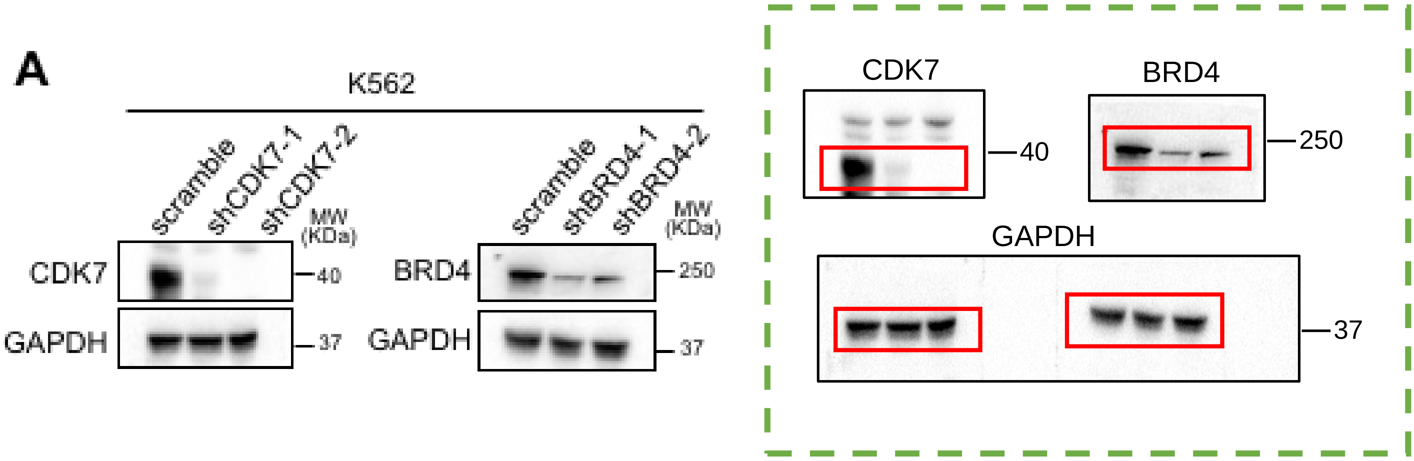

Figure S5B

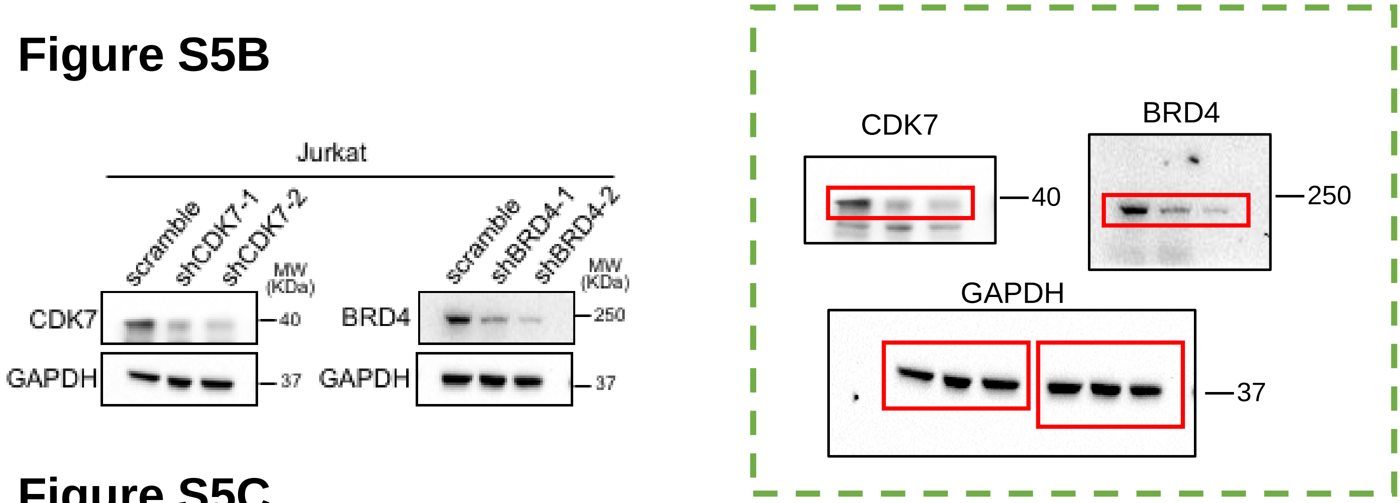

Figure S5C

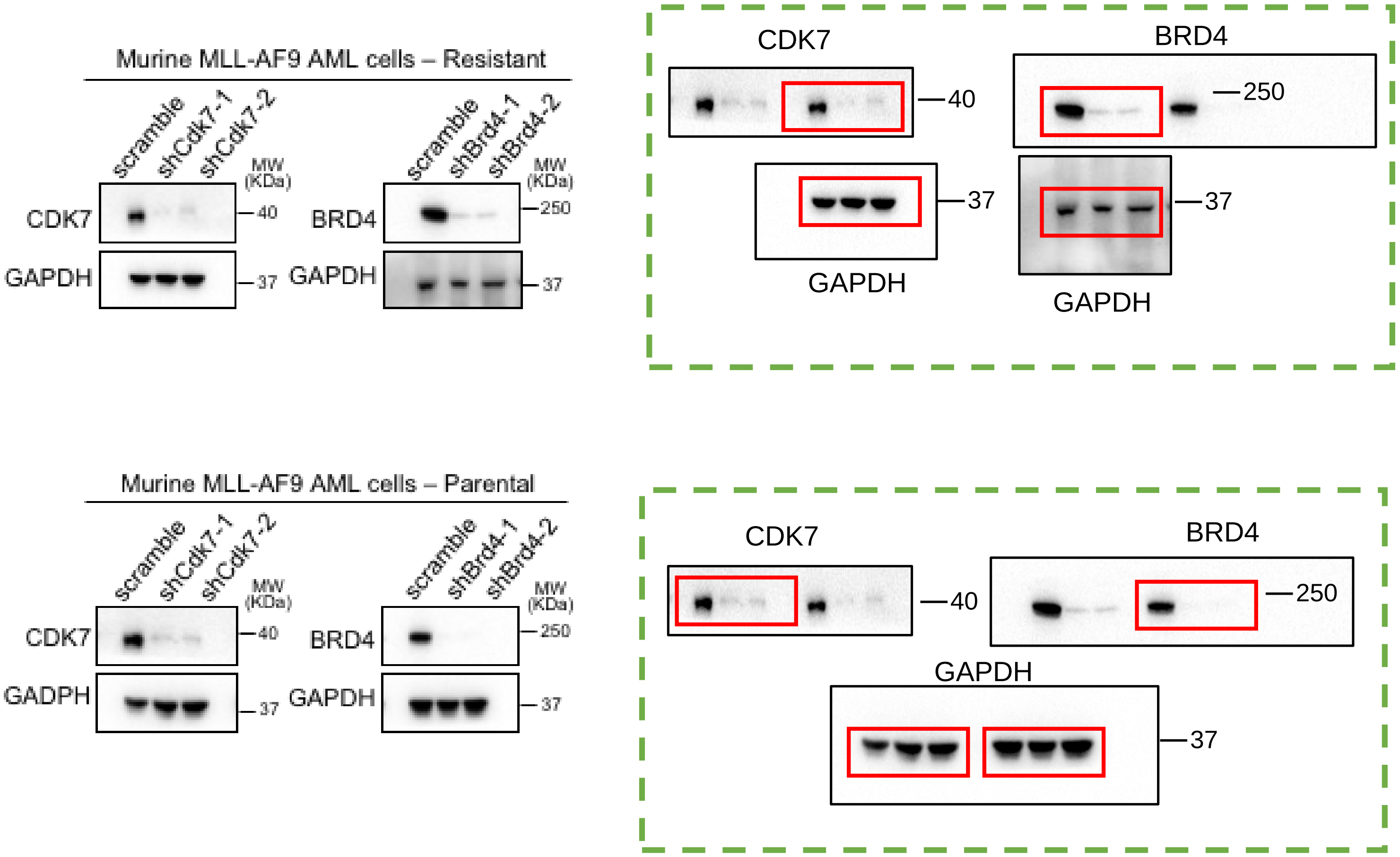

Figure S7A

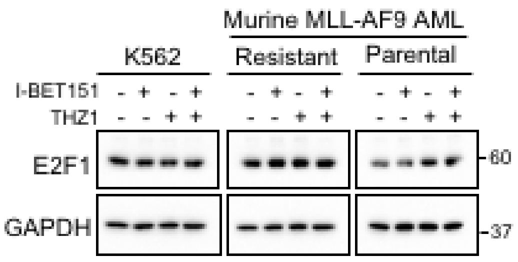
Murine MLL-AF9 AML
K562
Resistant
Parental
I-BET151 - + - + - + - + - + - +
THZ1 - - + + - - + + - - + +
E2F1
GAPDH
60
37

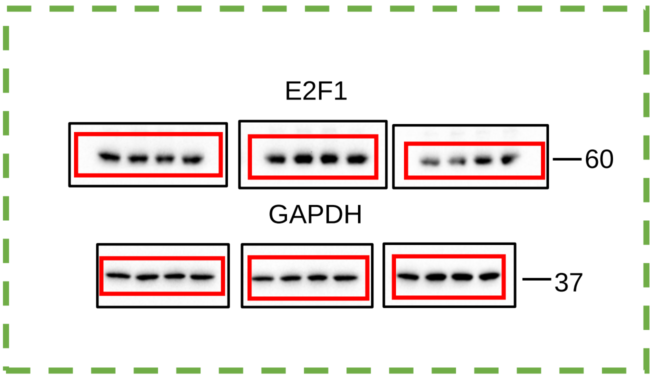
E2F1
60
GAPDH
37

Figure S7B

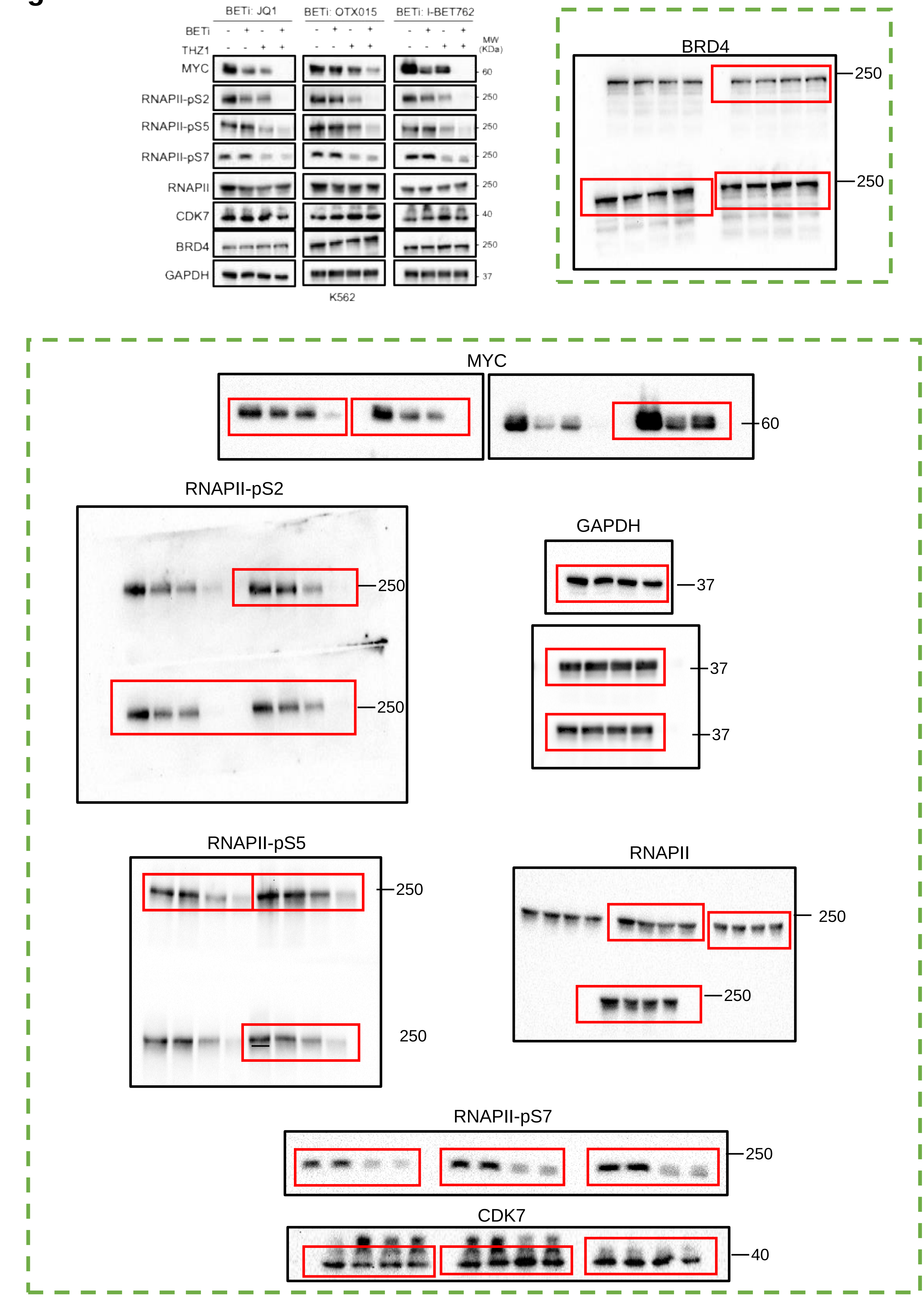

BETi: JQ1
BETi: OTX015
BETi: I-BET762
BETi
THZ1
MW (KDa)
MYC
RNAPII-pS2
RNAPII-pS5
RNAPII-pS7
RNAPII
CDK7
BRD4
GAPDH
60
250
40
37
K562
BRD4
250
250
MYC
60
RNAPII-pS2
250
250
GAPDH
37
37
37
RNAPII-pS5
250
250
RNAPII
250
250
RNAPII-pS7
250
CDK7
40

Figure S7C

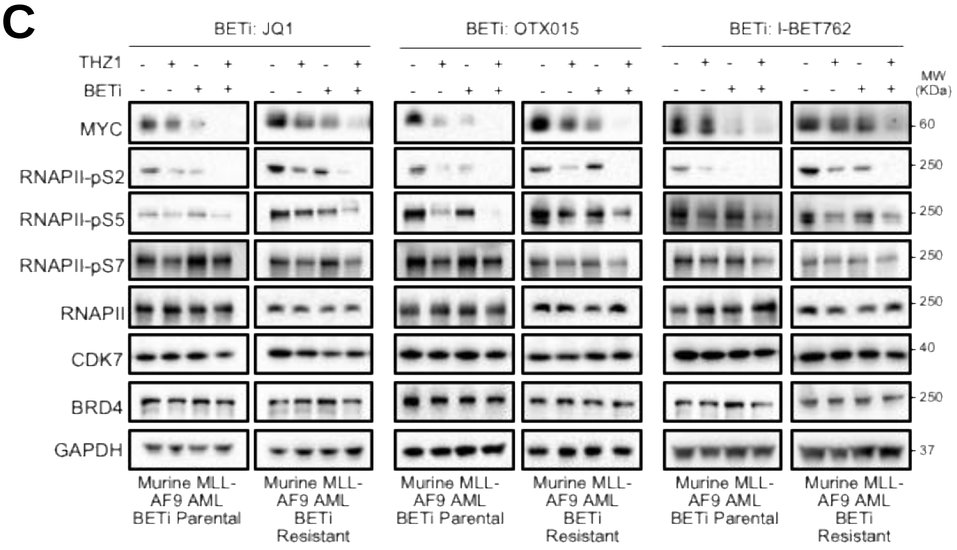

BETi: JQ1
BETi: OTX015
BETi: I-BET762
THZ1
BETi
MYC
RNAPII-pS2
RNAPII-pS5
RNAPII-pS7
RNAPII
CDK7
BRD4
GAPDH
MW (KDa)
60
250
40
37
Murine MLL-AF9 AML BETi Parental
Murine MLL-AF9 AML BETi Resistant

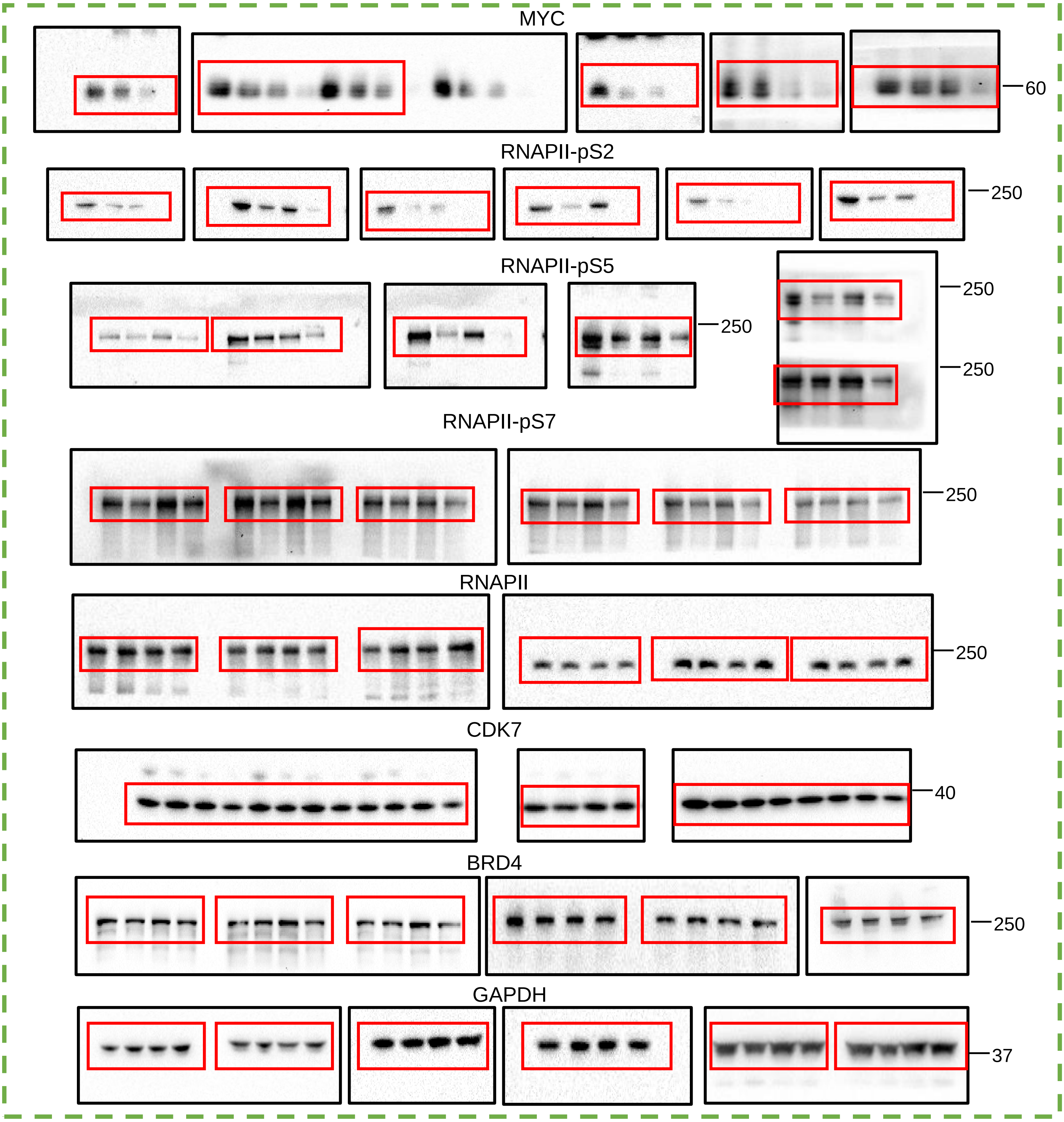

MYC
60
RNAPII-pS2
250
RNAPII-pS5
250
RNAPII-pS7
250
RNAPII
250
CDK7
40
BRD4
250
GAPDH
37

Figure S9B

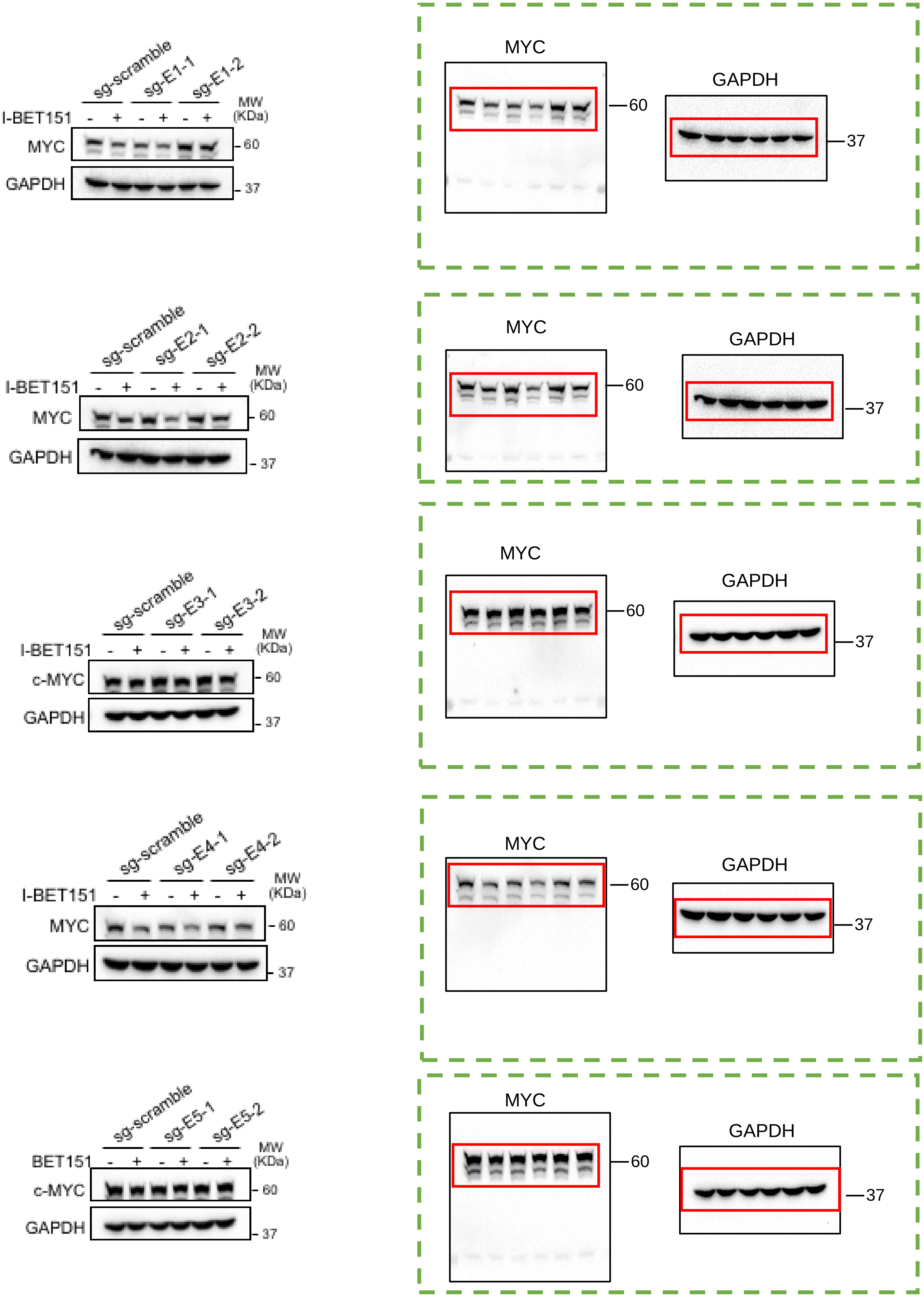

sg-scramble
sg-E1-1
sg-E1-2
I-BET151
- + - + - +
MW (KDa)
MYC
GAPDH
60
37
MYC
GAPDH
60
37
sg-scramble
sg-E2-1
sg-E2-2
I-BET151
MYC
GAPDH
sg-scramble
sg-E3-1
sg-E3-2
I-BET151
c-MYC
GAPDH
sg-scramble
sg-E4-1
sg-E4-2
I-BET151
MYC
GAPDH
sg-scramble
sg-E5-1
sg-E5-2
BET151
c-MYC
GAPDH

Figure S9H

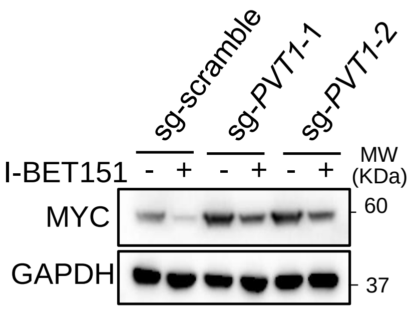

Murine MLL-AF9 AML parental

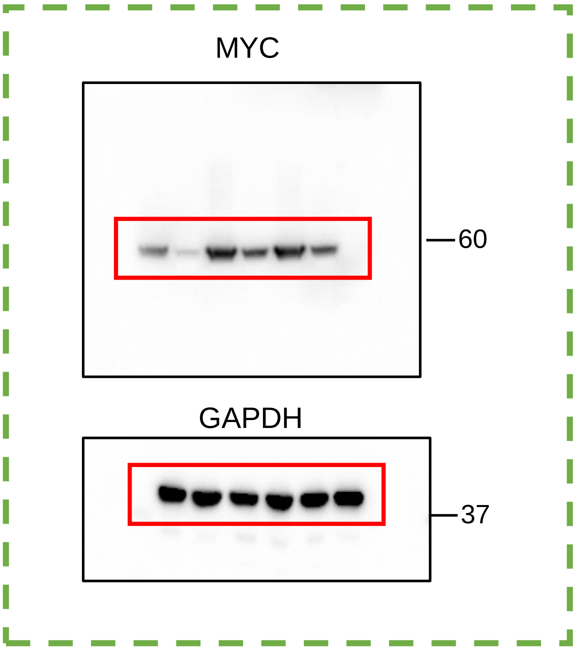

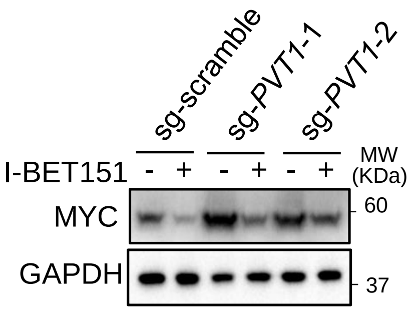

MOLM-13

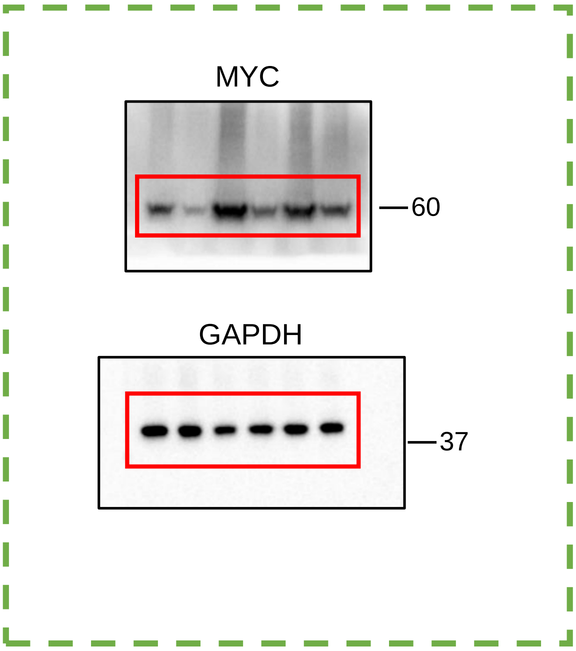

Supplement: Supplementary file 8 — Source Data [file 41467_2020_14604_MOESM8_ESM.zip › 214198_2_related_ms_4329106_q3hqn8.pdf]
